# Better antibodies, better science

**DOI:** 10.1371/journal.pbio.3004001

**Published:** 2026-10-07

**Authors:** Johan Duchêne

**Affiliations:** Institute for Cardiovascular Prevention (IPEK), Ludwig-Maximilians-University (LMU), LMU Open Science Center (OSC), German Center for Cardiovascular Research (DZHK), Munich, Germany

## Abstract

Poorly validated antibodies generate misleading results and waste research resources. This Primer explores two new studies in PLOS Biology that quantify the ethical costs of this issue, examine why unvalidated antibodies are still used, and identify solutions to reduce their use.

## Teaser

Poorly validated antibodies generate misleading results and waste research resources. Two new studies in *PLOS Biology* quantify the ethical costs of this issue, examine why unvalidated antibodies are still used, and identify solutions to reduce their use.

How would you know whether a protein is expressed in a specific cell type or whether its tissue expression is affected by a treatment? You would most probably order an antibody supposed to be directed against this protein, perform the experiment, obtain a beautiful signal, and draw conclusions. But how do you know that you have actually detected your protein of interest?

Antibodies are widely used in biomedical research to visualize proteins in cells and tissues, measure their abundance, and identify specific cell populations. Yet, obtaining a signal does not necessarily mean that the antibody specifically recognizes the protein of interest. An antibody may indeed bind other proteins. Several examples illustrate how antibodies can produce convincing signals while failing to reliably detect their intended protein targets [1–3]. Because the results can be biologically plausible, the problem may remain hidden until the antibody is independently challenged [1,2]. Alarmingly, a study found that more than 50% of tested antibodies were unfit for purpose in at least one of their potential applications [4]. The use of poorly validated antibodies can unfortunately lead to incorrect results, wasting research time and resources, and eventually contributing to the reproducibility crisis in biomedical research.

The problem is not new. Concerns about antibody specificity and validation have been raised for more than a decade [5,6], and the financial costs of unreliable antibodies were estimated in 2015 at $800 million wasted worldwide each year [6]. Several initiatives have sought to improve antibody validation. For instance, the International Working Group for Antibody Validation (IWGAV) developed recommendations for validating antibodies in common research applications and proposed five “pillars” of antibody validation, emphasizing that validation should be application-specific [7]. Yet, despite growing awareness of the problem, insufficiently validated antibodies remain widely used. A recent feature described how poorly performing antibodies continue to affect biomedical research [3]. The article also highlighted new initiatives seeking to improve antibody characterization and access to validation data. One such initiative, YCharOS (Antibody Characterization through Open Science), systematically tests commercially available antibodies using knockout samples [8], the gold-standard approach for assessing antibody specificity. A signal in knockout samples indicates that the antibody binds proteins other than the intended target. By making such validation data available, YCharOS could shift antibody validation from an individual laboratory’s troubleshooting exercise towards a shared resource for the research community.

Despite growing recognition of the problem and efforts to address it, why does it persist? Why has antibody selection and validation practice changed so little, and what would it take to reduce the use of antibodies for which performance has not been adequately validated?

Two complementary studies in this issue of PLOS Biology [9,10] now address these questions. The first study, by Biddle and colleagues, quantifies the problem [9]. The authors used data from the YCharOS platform to investigate the ethical consequences of insufficient antibody validation. Among 614 antibodies characterized across multiple applications, the authors identified 97 that failed under all tested conditions. They analyzed 785 publications in which these antibodies had been used and identified thousands of animal and human biological samples studied without reported evidence of validation. Extrapolating from these observations, they estimated that millions of animal and human tissue samples may have been used in experiments involving antibodies that would fail independent testing, highlighting the potential ethical consequences of inadequate validation. The study also explored why the problem persists, finding that antibody selection by surveyed scientists was often skewed by previous use, published literature, and supplier reputation rather than systematically evaluated for its performance.

Identifying a problem is important, providing solutions even more so. This is where the second study, by Blades and colleagues, comes in [10]. The problem cannot be solved simply by asking researchers to validate their antibodies more rigorously. Moreover, responsibility is distributed across multiple actors, with each potentially relying on others to address it. The study brought together these different stakeholders to identify practical and achievable solutions. The authors conducted a Delphi consensus process involving 32 international experts representing five stakeholder groups: academic researchers, scientific publishers, research funders, antibody manufacturers, and institutional research leaders. Out of 33 proposed actions, the participants identified 15 which could both effectively and feasibly be implemented by 2030. Importantly, these distribute responsibility across the research ecosystem. Researchers need access to appropriate training and validation resources, while institutions, funders, publishers, and antibody manufacturers can influence how antibodies are selected, validated, reported, and documented. The study therefore moves beyond the general recommendation that “antibodies should be better validated” towards a roadmap in which different stakeholders have distinct roles in improving antibody reliability.

Whether this roadmap will translate into changes in practice remains an important question. Consensus among stakeholders is a necessary first step, but implementation will require sustained commitment and coordination across the research ecosystem. The next challenge is to make these solutions part of routine biomedical research. This challenge may become even more important as new technologies reshape how biomedical research is conducted. AI is increasingly used to search and synthesize the scientific literature but lacks critical thinking and does not exclude findings based on poorly validated reagents making it potentially unreliable. Such findings could then be propagated into new analyses, recommendations, and hypotheses. It is crucial that future AI tools are trained to take this issue into account. At the same time, AI is being used to design new protein binders that could complement or replace conventional antibodies [11,12]. The success of these new design strategies will also rely on whether lessons learnt from antibody validation are taken into consideration and on systematic testing of their ability to specifically recognize the intended target.

Overall, the scientific community needs evidence that the reagents used to generate biomedical findings actually measure what researchers intend them to. We should therefore build rigorous validation into routine research practice, supported by the wider research ecosystem. After all, better validated reagents enable better science.
